# Mitochondria-Targeted Nanocarriers Promote Highly Efficient Cancer Therapy: A Review

**DOI:** 10.3389/fbioe.2021.784602

**Published:** 2021-11-12

**Authors:** Zeng Zeng, Chao Fang, Ying Zhang, Cong-Xian Chen, Yi-Feng Zhang, Kun Zhang

**Affiliations:** ^1^ Department of Medical Ultrasound, Zhejiang Provincial People’s Hospital, Hangzhou, China; ^2^ Department of Medical Ultrasound and Central Laboratory, Shanghai Tenth People’s Hospital, Tongji University School of Medicine, Shanghai, China

**Keywords:** mitochondria, nanocarriers, phototherapy, chemotherapy, combined immunotherapy

## Abstract

Mitochondria are the primary organelles which can produce adenosine triphosphate (ATP). They play vital roles in maintaining normal functions. They also regulated apoptotic pathways of cancer cells. Given that, designing therapeutic agents that precisely target mitochondria is of great importance for cancer treatment. Nanocarriers can combine the mitochondria with other therapeutic modalities in cancer treatment, thus showing great potential to cancer therapy in the past few years. Herein, we summarized lipophilic cation- and peptide-based nanosystems for mitochondria targeting. This review described how mitochondria-targeted nanocarriers promoted highly efficient cancer treatment in photodynamic therapy (PDT), chemotherapy, combined immunotherapy, and sonodynamic therapy (SDT). We further discussed mitochondria-targeted nanocarriers’ major challenges and future prospects in clinical cancer treatment.

## Introduction

Cancer is a threat to human beings, and the incidence and mortality rate are rising nowadays. It can be described like a state of multifaceted cellular deregulation which involves bioenergetic regulations and proliferation (Dong and Neuzil, 2014). It is extremely important to find effective ways against various cancers (Yang et al., 2020). There are many traditional therapies for cancers such as surgery, radiotherapy, and chemotherapy (Dolmans et al., 2003). New methods such as immunotherapy have been used recently (Kelland, 2007). Mitochondria’s role in cancer has also been widely recognized in the last 10 years (Lu et al., 2016).

Otto Warburg observed that mitochondria were dysfunctional in cancer cells (Warburg, 1956). Because of the central role of mitochondria, they were called “culprits” for the malignancy of cancer cells. Nowadays, mitochondria serve as a potential target for cancer therapeutics. They are dynamic eukaryotic organelles which control metabolic activities and vital functions of cells. Mitochondria produce adenosine triphosphate (ATP) for cell survival; they also control lethal functions of cells, such as necrosis and apoptosis (Kroemer, 2003). Mitochondria-targeted therapeutic agents can play in the central point of cells. So it is an efficient way of leading the therapeutic agent to the mitochondria in eliminating cancer cells.

Mitochondria are the cells’ powerhouses, maintaining cells’ lives and playing a vital role in regulating their death, which occur on their membranes upon permeabilization (Armstrong, 2006; Ubah and Wallace, 2014). Around 1995, mitochondria not only were regarded as an area for energy production but also controlled cell death regulation (Ubah and Wallace, 2014). Once mitochondrial membrane permeabilization (MMP) happens, cells will die *via* various death pathways such as necrosis or apoptosis. Mitochondrial dysfunction such as increasing oxidative stress and deregulation of apoptosis and/or impaired oxidative phosphorylation plays a vital role in the pathophysiological mechanism. They also control the pathogenesis of other acquired pathologies and congenital anomalies which include cancer and other diseases (Ferrin et al., 2011; Serviddio et al., 2011; Victor et al., 2011).

Mitochondria play an important role in the regulation of ROS production, bioenergetics, and apoptotic cell death, thus having a targeting ability as well as therapeutic benefits (Ubah and Wallace, 2014). They function as targeting subcellular organelles in the treatment of many diseases and attract attention in the research community of medicine and biology significantly. Many ways have already been developed to deliver kinds of drugs to mitochondria. Under normal conditions, ROS is needed for signaling. When apoptosis is inhibited in cancer cells, ROS helps in the neoplastic transformation. What is more, in order to support cancer cells’ survival in harsh tumorigenic conditions, for example hypoxia and nutrient depletion, mitochondria will provide flexibility through several ways either by up- or downregulation (Wallace, 2012).

**FIGURE 1:**
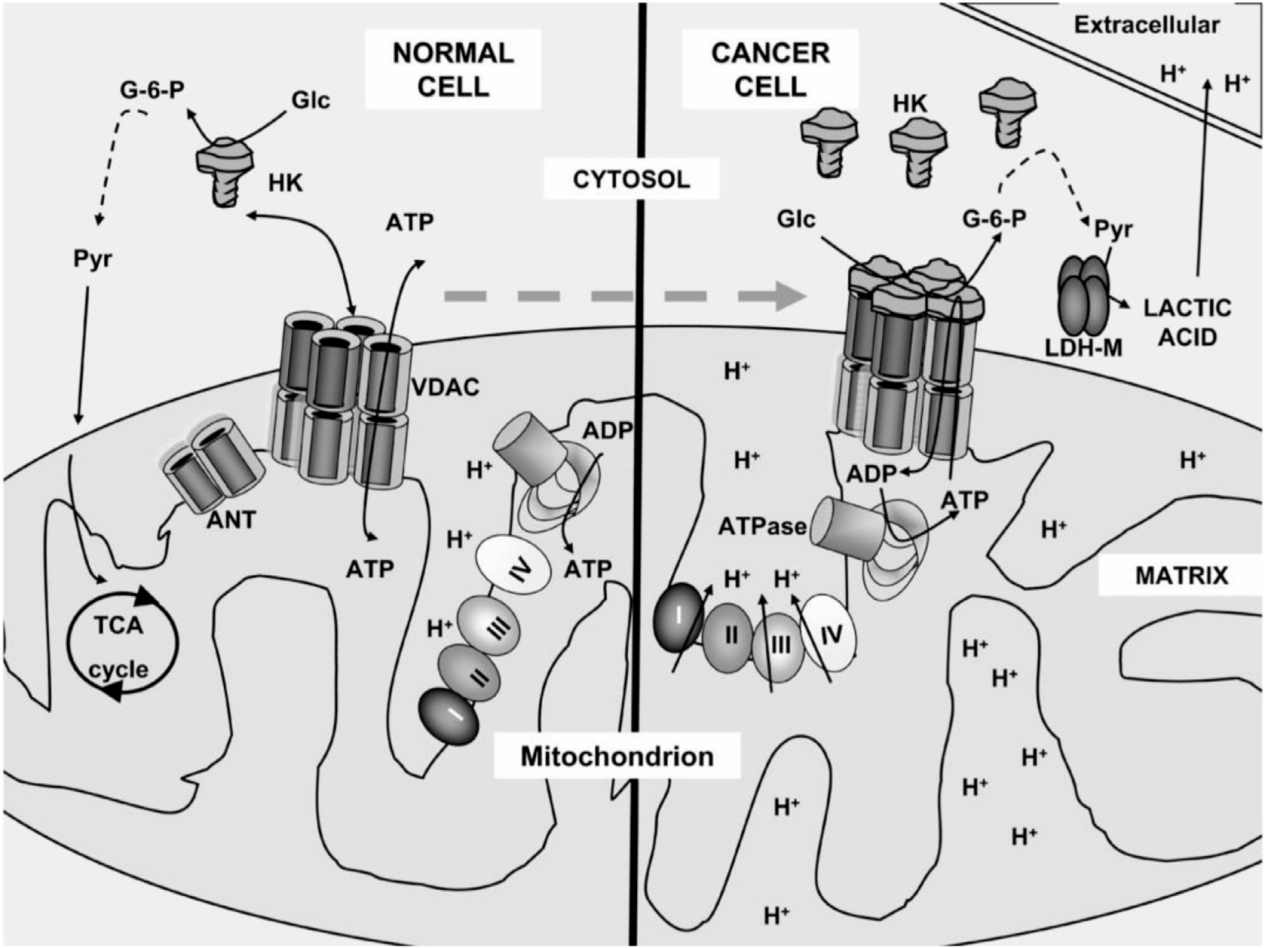
Mitochondrial metabolism of a normal cell and a cancer cell. In the normal cell, pyruvate was carried into the mitochondria and converted into the tricarboxylic acid cycle. However, in the cancer cell, pyruvate did not enter into the mitochondria as it converted to lactic acid and acidified the extracellular milieu (Ralph and Neuzil, 2009).

Healthy and cancerous mitochondria are different in structure and function, such as energy production pathway, respiratory rate, membrane potential, and gene mutations (Figure 1) (Gogvadze et al., 2008; Ralph and Neuzil, 2009; Jeena et al., 2019). In healthy normal cells, mitochondria control the regulation of various functions to keep the growth and death cycle of cells (Kroemer, 2006). While in cancer cells, dysregulation of mitochondrial metabolism always occurs because of higher metabolic demand with rapidly proliferating cells (Wisnovsky et al., 2016). Cancer cells have extensive metabolic reprogramming. They are much susceptible to mitochondrial perturbations than some healthy cells (Nadege et al., 2009). Owing to that, mitochondria can be used for the designation of selectively targeting systems for treating cancer.

Recently, nanomedicine has become popular in treating cancer. Nanoparticles have characters of small size, high versatility, high surface-volume ratio, and stability *in vivo* (Wicki et al., 2015). Mitochondria-targeted carriers such as nanoparticles and liposomes are active molecules. They can be delivered instead of being directed to the mitochondria selectively. The research area of cancer-selective carriers and nanoparticles is highly active (Biasutto et al., 2010). Transporters are required for macromolecules and small-molecule drugs during cellular internalization, reducing the burden on targeting the mitochondria of cancer cells selectively to the transporters (Ubah and Wallace, 2014). It is a major obstacle because of the elevating clearance rate by the reticuloendothelial system (RES), and other organs, nanoparticles, and liposomes will eliminate in a rapid speed, thus limiting the use of nanoparticles and liposomes in cancer therapy (Torchilin, 2005). However, recent research found that carrier size reduction to below 200 nm will allow for accumulating in cancer cells efficiently because of permeability and retention effect enhancement (Biasutto et al., 2010). In our review, we discuss about the progress in mitochondria-targeted-nanocarrier cancer therapy in many aspects such as PDT, chemotherapy, combined immunotherapy, and sonodynamic therapy.

## Mitochondria and Mitochondria-Targeted Nanocarriers

### Introduction of Mitochondria-Targeted Multifunctional Nanoparticles

Mitochondria contain the inner mitochondrial membrane, intermembrane space, and outer mitochondrial membrane (Friedman and Nunnari, 2014). Each membrane has a distinct protein population (Porporato et al., 2018). Mitochondria are energy-producing structures and play the major part for cells’ aerobic respiration (Bhandary et al., 2012). Thus, mitochondria are called the “powerhouse of the cell” (Peixoto et al., 2010). They play important roles in apoptosis regulation, cell signaling, and energy metabolism in drug-induced cancer cell death and are thus considered targets in cancer chemotherapy (Grad et al., 2001). Many scholars have reviewed the development of chemotherapeutic drugs for mitochondria in fighting cancer (Costantini et al., 2000; Wen et al., 2013; Wu et al., 2018a).

Cancer cells have rapid proliferation and need more mitochondria. Mitochondria play a vital role in the energy metabolism and regulation of the cell cycle. It is also known that mitochondria play an important role in triggering cell death and complex apoptotic mechanisms through several mechanisms which include release or activation of proteins, energy metabolism, and disruption of electron transport (Hiendleder et al., 1999; Waterhouse et al., 2001; Gulbins et al., 2003). MMP is the critical point leading to programmed cell death. MMP is under the control of the permeability transition pore complex (mPTPC), which is a multiprotein complex that is formed at the contact position between the inner membranes and outer membranes of mitochondria. Apoptosis controls tissue homeostasis, while inhibition of apoptosis helps in the changeable process of normal cells to cancer cells (Costantini et al., 2000). Most types of cancer are linked with the dysfunction of apoptosis (Kaufmann and Gores, 2000; D'Souza and Weissig, 2004). Cancer cells are in favor of the glycolytic process even under aerobic conditions for the source of ATP. Adaptations often result in changing mitochondrial function which includes mutations in mitochondrial DNA (mtDNA) (Carew and Huang, 2002). Thus, mitochondria are described as a “prime target” for pharmacological intervention (Szewczyk and Wojtczak, 2002).

**FIGURE 2:**
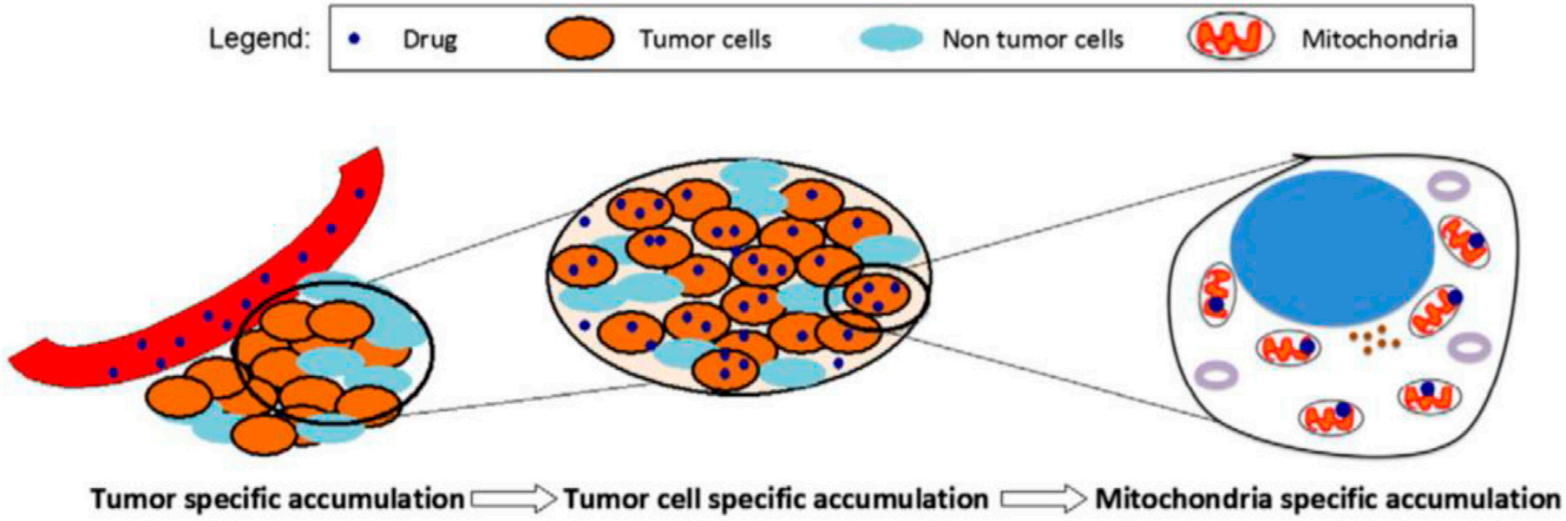
A schematic representing the mitochondria-specific targeting strategy (D'Souza et al., 2011).

In Figure 2, the approach of selecting accumulation to targeting tumor mitochondria was underlined, wherein a two-step accumulation process is needed. The first one is intratumoral drug accumulation, and the other is drug accumulation in mitochondria (D'Souza et al., 2011).

**FIGURE 3:**
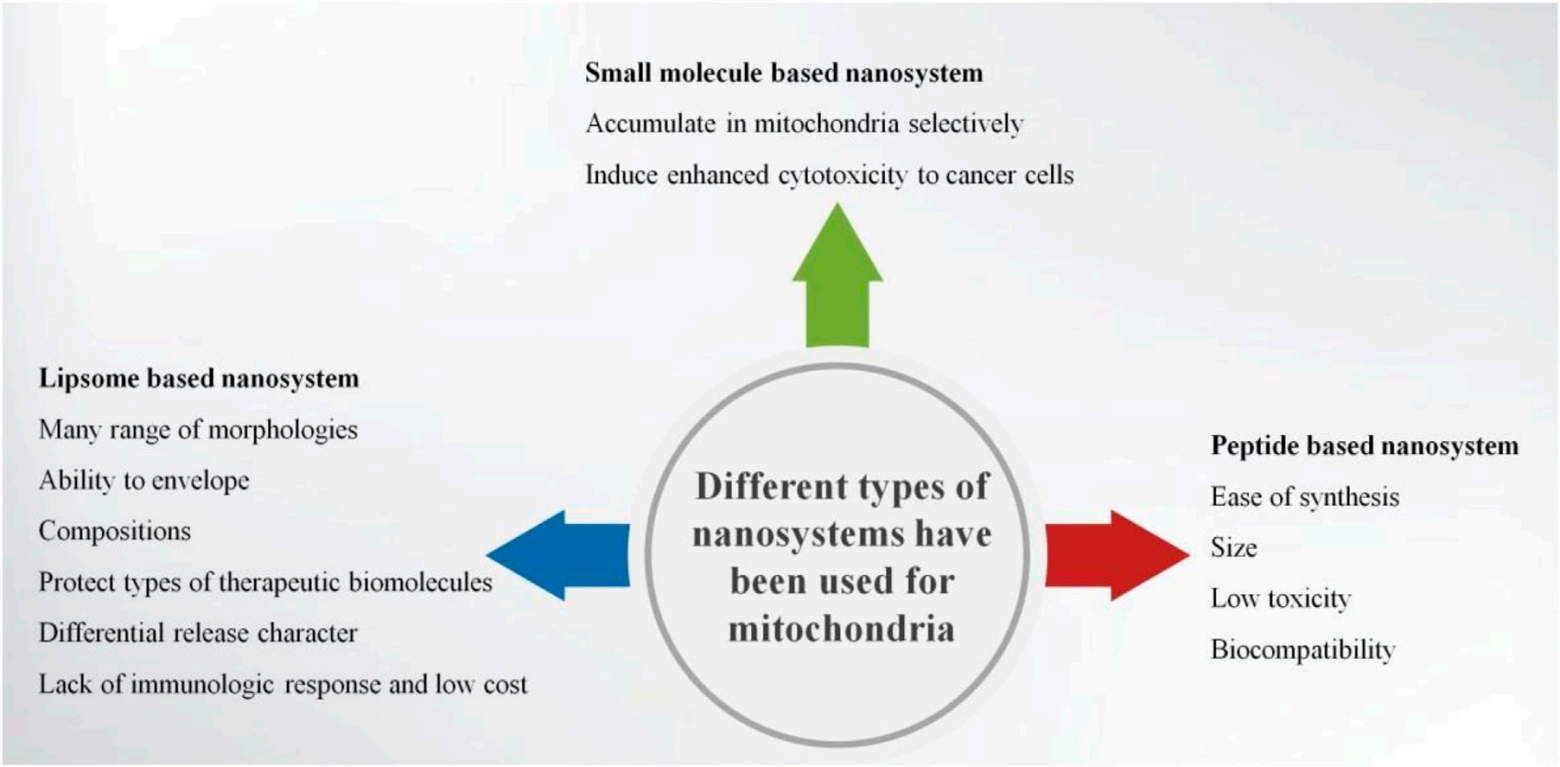
Diagram of the different types of nanosystems for targeting mitochondria.

Nanomaterials are good tools for diagnosis, targeted therapy, and molecular imaging. Targeting, imaging, therapeutics, and other multiple functionalities could be integrated into one nanoparticle (Zhang et al., 2011). Nanocarriers such as liposomes, micelles, and solid nanoparticles behave in a non-chemical way to modify the disposition of drug molecules. A nanocarrier system loaded with some drugs can afford targeted delivery. Most of the nanocarriers can be additionally modified in order to target to specific tissues or specific cells and afford cell-specific recognition (Torchilin, 2007; Ganta et al., 2008; Mishra et al., 2010). Enhanced permeability and retention (EPR) effect can help nanoparticles passively target the place of leaky vasculatures (Hatakeyama et al., 2007; Ma et al., 2009; Nallamothu et al., 2006). Nanocarriers can affect the drug accumulation of tumor and mediate the accumulation of mitochondria within tumor cells (D'Souza et al., 2011). Thus, mitochondria-targeted anticancer approaches can be used in clinic. Nucleic acids, antioxidants, anticancer agents, and proteins can be delivered into nanostructures through mitochondrial targeting of cancer cells (Zhang et al., 2011). Examples such as small-molecule-based nanosystems, peptide-based nanosystems, and liposome-based nanosystems had been successfully used in mitochondrial targeting. These nanosystems were widely used in targeting cancer cells, especially to the mitochondria of cancer cells (Figure 3).

Based on mitochondria’s redox balancing, involvement in bioenergetics, and regulation of several cell survival or death pathways, it is reasonable to target the mitochondria for therapeutic benefit (Ubah and Wallace, 2014). Mitochondria-targeting drug delivery shows value in cancer treatment. The interior negative mitochondrial transmembrane potential is 130–150 mV (Weissig and Torchilin, 2001). Through directly attaching delocalized lipophilic cations to nanocarriers or drug molecules, mitochondria-targeting drug delivery can be achieved (Hu et al., 2014).

Triphenylphosphonium (TPP) always acts like a mitochondrial targeting ligand and can be taken by the mitochondrial membrane. It is a small molecule which can be used primarily for mitochondrial targeting (Patil et al., 2019). Although TPP is the most used mitochondrion tropic ligand and is able to deliver cargos to the mitochondria, the targeted drug delivery of TPP derivatives is limited due to its rapid clearance in circulation (Mo et al., 2012; Marrache et al., 2014; Yue et al., 2016). A recent study showed that PEGylation is the most used strategy and is responsible for nanoparticle stealth from the reticuloendothelial system. It improved the stability and resulted in an enhanced accumulation in tumor tissue *via* improving EPR effect. However, PEGylation’s shielding effect can prevent the cellular uptake of the NPs (Han et al., 2015). Other small molecules such as guanidine, berberine, and rhodamine can also target to mitochondria.

Ma et al. (2018) revealed that an interparticle plasmonic coupling effect activated nanoevents which cause hyperthermia in mitochondria to strike tumor cells selectively and not damage adjacent normal cells. Avoiding damage to adjacent normal cells is extremely important especially in brain tumor. This mitochondria-templated accumulation strategy could provide an effective model in striking tumor and protecting adjacent normal tissue.

### Lipophilic Cations-Based Mitochondria-Targeted Nanocarriers

At the beginning of the 1960s, liposomes were discovered, and in the 1970s they were proposed as a drug carrier system (Bangham et al., 1965a; Bangham et al., 1965b). Liposomes are currently considered as the archetype of all pharmaceutical nanocarriers. These nanovesicles can sequester lipophilic drugs in their phospholipid bilayer membranes and hydrophilic drug molecules in their aqueous inner space (Weissig, 2012). Liposome-based systems have the ability to deliver agents to the mitochondria and treat cancer. Using liposomes as a vehicle has many advantages in drug delivery such as many ranges of morphologies, ability to envelope, compositions, protection of types of therapeutic biomolecules, differential release character, lack of immunologic response, and low cost (Tros de Ilarduya et al., 2010). Kawamura et al. (2020) developed the MITO-Porter system which can be used to deliver genes, proteins, nucleic acids, and small molecules to the mitochondria specifically through membrane fusion.

### Peptides-Based Mitochondria-Targeted Nanocarriers

Because of ease of synthesis, size, low toxicity, and biocompatibility, peptides have the potential of being mitochondria-targeting ligands (Wu et al., 2018b). The peptide should have optimum positive charge and hydrophobicity to penetrate the mitochondrial membrane (Horton et al., 2008). Three types of peptides are widely used in constructing mitochondria-targeting nanosystems, such as mitochondria-targeting signal peptides (MTSs), mitochondria-penetrating peptides (MPPs), and Szeto-Schiller (SS) (Qin et al., 2021).

**FIGURE 4:**
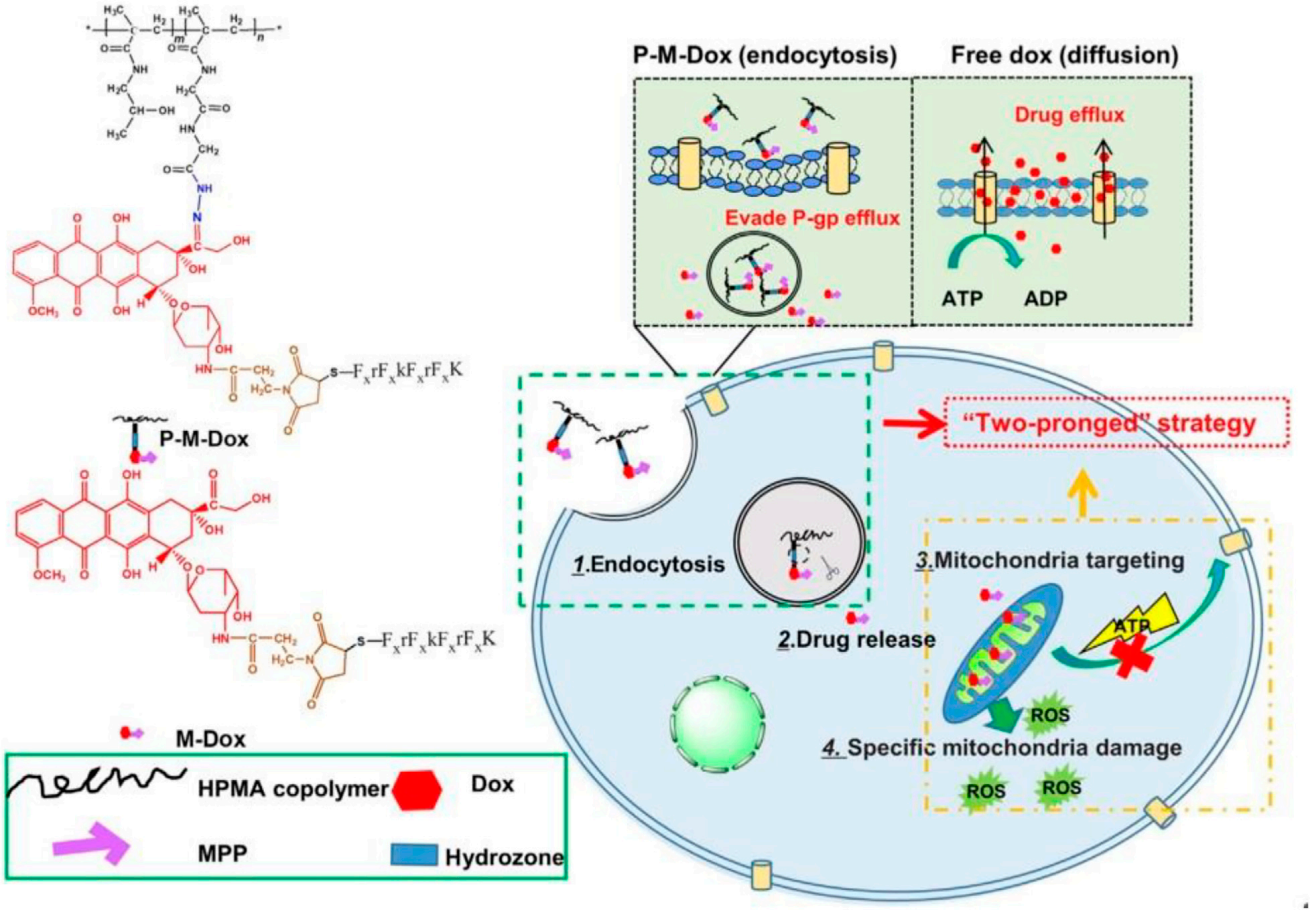
P-M-Dox overcomes multidrug resistance through simultaneous drug-efflux circumvention and mitochondrial targeting (Zhou et al., 2019).

Zhou et al. (2019) synthesized Dox modified with mitochondrial membrane-penetrating peptide (MPP) which is combined with (HPMA) copolymers (P-M-Dox) and provided it as a promising way to deal with cancer which is drug-resistant by drug efflux circumvention simultaneously and mitochondrial delivery directly (Figure 4).

MTSs enter the mitochondrion through tightening the mitochondrial import machinery on the outer mitochondrial membrane. However, MTSs are too insoluble to cross the plasma membrane, limiting their intracellular applications (Wu et al., 2018b). Lindgren et al. (2000) combined cell-penetrating peptides (CPPs) and MTS to serve as cell-permeable mitochondrial targeting peptides which can deliver agents. Lin et al. (2015) utilized MTS–CPP successfully for the mitochondrial delivery of nucleic acids and proteins.

The SS peptide is made of four positively charged amino acids. Due to the antioxidant effect of SS peptides, they can be carrier components in treating mitochondria-related diseases (Dai et al., 2011). The newly established amphiphilic mitochondria-targeting chimeric peptide drug delivery system (DDS) can overcome drug resistance (Han et al., 2016). Han et al. (2016) found that chimeric peptides can encapsulate doxorubicin and target to tumor mitochondria in *in vitro* studies. DDS could control the release of doxorubicin and help in PDT in mitochondria. Although drug resistance is a big obstacle in traditional chemotherapy, the DDS strategy gave a new way to overcome it.

## Mitochondria-Targeting Nanosystems for Cancer Therapy

### Mitochondria-Targeted Nanocarriers in PDT

Compared with conventional therapeutic strategies for cancer treatment, PDT has characteristics of high selectivity, rapid action, and no severe side effects (Hilf, 2007; Yang et al., 2019). PDT is a safe treatment which relies on oxygen to produce cytotoxic ROS under visible light and photosensitizers (PS) in cells (Castano et al., 2006). PS can combine together to induce cancer cell death (Jeena et al., 2019). Under light irradiation, PS can be excited and can transfer energy to molecular oxygen to generate ROS. In the tumor microenvironment, oxygen (O_2_) can convert into singlet oxygen (^1^O_2_) and cause damage to cancer cells (Ethirajan et al., 2011). All these procedures occur in the area where the light is irradiated particularly. Thus, PDT agents can cause less bad effects than other conventional drugs.

However, there exists a barrier for PDT of behaving actively in the cancer area. The tumor microenvironment is always hypoxic, thus hampering the production of toxic singlet oxygen. Inhibition of mitochondrial respiration can increase the production of intra-mitochondrial oxygen, thus enhancing the efficiency of PDT. Therefore, PDT becomes hotter if mitochondria are targeted compared with subcellular targets or any other cells. PDT agents can be modified with metal complexes which have lipophilic cations, IR-780-based PS, or cyanine (Jeena et al., 2019). Combination of PS with cationic peptides is the most common adopted method to direct the PS inside the mitochondria of the cell.

Mitochondria-targeted PS behave with thousand times efficacy than those localized in the extracellular matrix or the cell membranes (Saneesh Babu et al., 2017). A hollow silica lattice structure which was based on multistage DDS combined with encapsulated catalase and chlorine e6 (Ce6) (a photosensitizing agent) was utilized representatively (Yang et al., 2018). Combined with programmed death-ligand 1 (PD-L1), this nanosystem can improve PDT efficacy and enhance the infiltration of cytotoxic T lymphocytes (CTLs) into tumors, indicating the metastasis of cancer and potent inhibition. Glycolysis inhibition can lead to compensatory activation of their oxidative phosphorylation in cancer cells (Qin et al., 2021). Cutting off the energy supply to realize the simultaneous inhibition of both oxidative phosphorylation and glycolysis is the most direct strategy for cancer treatment.

**FIGURE 5:**
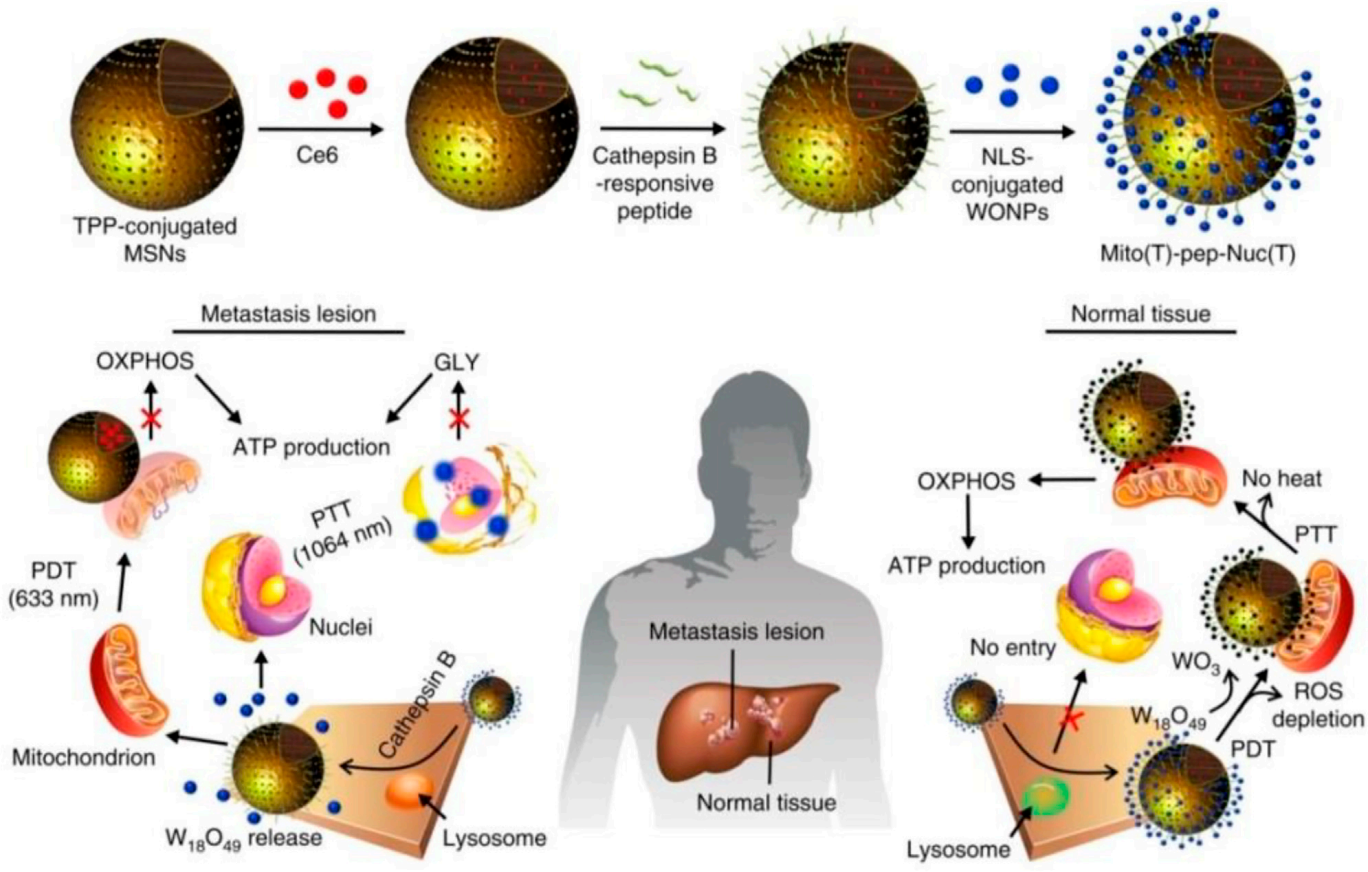
Working principle of Mito (T)-pep-Nuc (T) (Huo et al., 2019).

Huo et al. (2019) established a system which consists of photosensitizer (Ce6)-encapsulated mesoporous silica nanoparticles (MSNs) and W_18_O_49_ nanoparticles (WONPs) (Figure 5). The overexpressing cathepsin B cleaved peptide linkers and can allow WONPs and MSNs to target the nucleus and mitochondria in cancer cells, respectively. Then, laser irradiation was applied in order to trigger PDT which was mediated by Ce6 and WONPs. At last, this strategy could damage both the nucleus and mitochondria, cutting off the energy supply.

### Mitochondria-Targeted Nanocarriers in Chemotherapy

Chemotherapy is extraordinarily critical in systemic therapy of cancer therapy. Chemotherapeutics such as doxorubicin (Dox), cisplatin (Pt), and their combinations are commonly used in cancer therapy (Zheng et al., 2014). However, chemotherapy has its own shortcomings such as drug resistance of cancer cells, low-targeting selectivity to malignant areas, and some adverse side effects to healthy tissues (Xue et al., 2012). Thus, it is significant to circumvent obstacles and improve the efficiency of chemotherapy. There are many nanosized chemotherapeutic formulations, which include liposomes, polymeric micelles, and albumin NPs which have been used in different stages of clinical trial (Mehra et al., 2015). For example, Abraxane and Doxil have been demonstrated to improve the patients’ safety and decrease the toxic side effects.

Mitochondria-targeted anticancer agents can conjugate mitochondria-targeting moieties, such as TPP, cationic peptides, or pyridinium, with anticancer drugs such as doxorubicin, chlorambucil, cisplatin, and camptothecin (Jeena et al., 2019).

**FIGURE 6:**
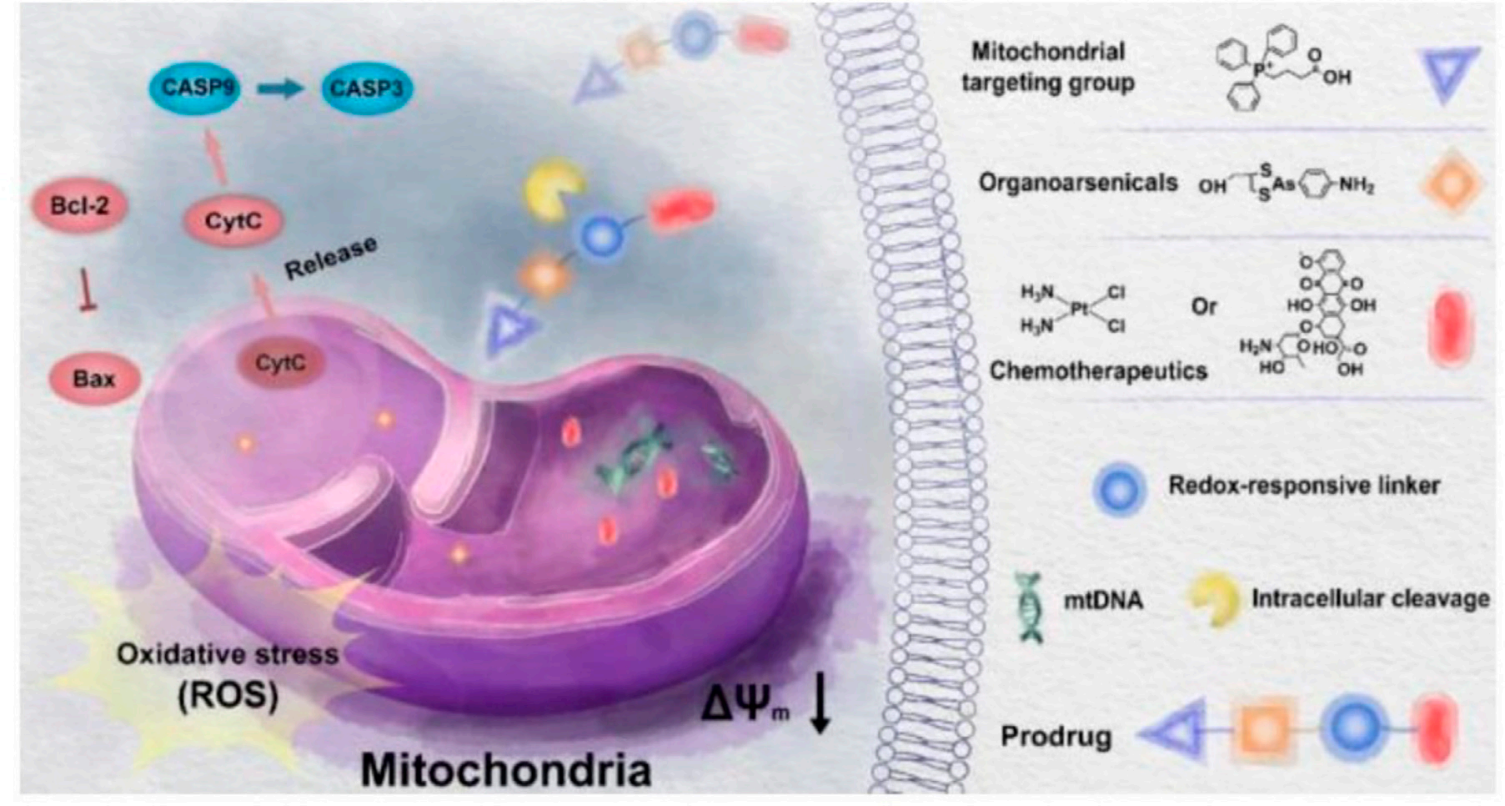
Illustration mechanism of underlying mitochondria targeting organoarsenic prodrugs for bioenergetic cancer therapy (Luo et al., 2021).

TPP is known as a mitochondrial targeting ligand. Studies showed that doxorubicin (Dox) and TPP-linked cisplatin (Pt) can disrupt mitochondrial DNA (mtDNA), raising the levels of the mitochondrial reactive oxygen species (mtROS) and leading to mitochondrial dysfunction (Jin et al., 2018; Babak et al., 2019). However, some anticancer effects cannot be achieved by delivering traditional drugs to mitochondria (Luo et al., 2021). Luo et al. (2021) reported new activatable mitochondria targeting organoarsenic prodrugs by incorporating traditional DNA targeting chemotherapy drugs with mitochondria-targeting organoarsenicals through cleavable linkers for treating cancer effectively (Figure 6). Under the help of the TPP-targeting group, prodrugs can accumulate in the mitochondria selectively. The prodrugs were able to release trivalent organoarsenicals and chemotherapeutics upon reduction, leading to mitochondria-mediated apoptosis in cancer (Luo et al., 2021).

Han et al. (2015) established a self-delivery system PpIX-PEG-(KLAKLAK)_2_ which was designated as PPK. PPK has a high drug loading ability and capacity in reactive oxygen species. The *in situ* generation of reactive oxygen species in mitochondria could enhance PDT efficacy through a long-time irradiation, thus leading to cell death and decrease in mitochondrial membrane potential. They demonstrated that PPK with a dual-stage light irradiation can be a good nanoplatform to treat cancer.

### Mitochondria-Targeted Nanocarriers in Combined Immunotherapy

**FIGURE 7:**
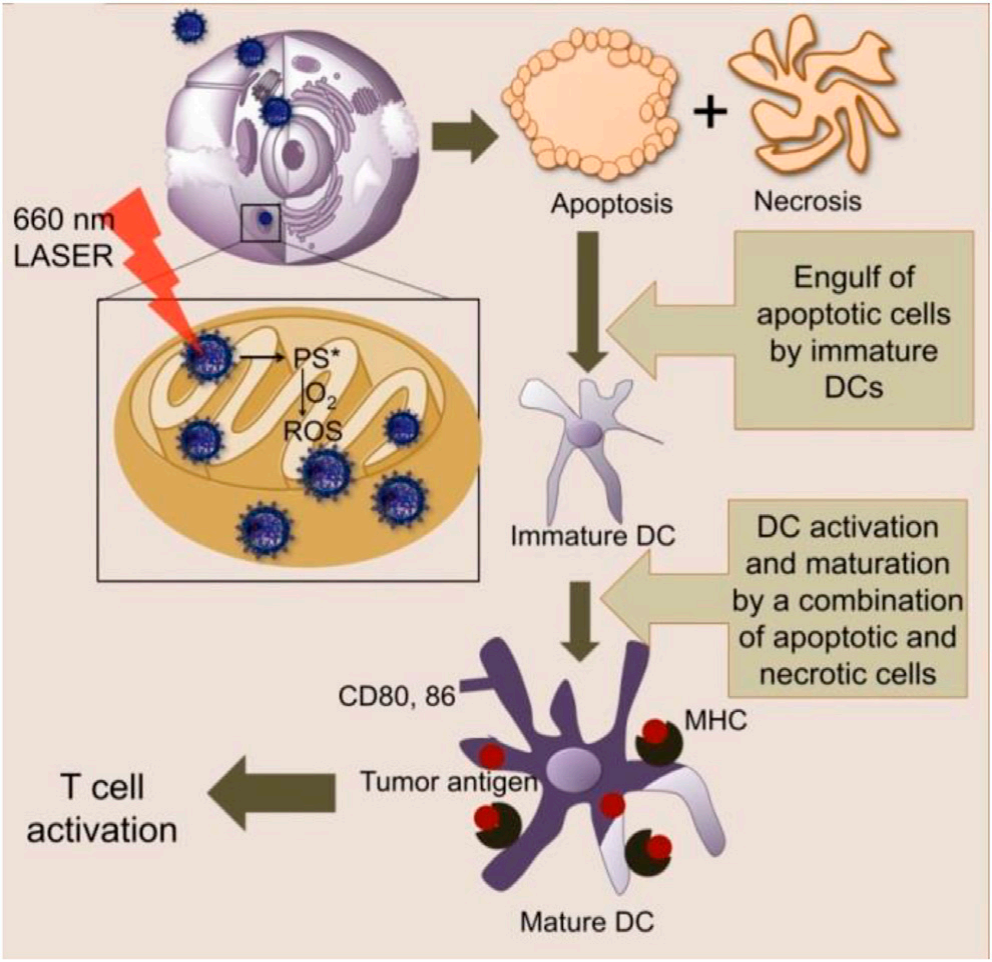
Mitochondria-targeted NPs act upon light activation inside the mitochondria to produce ROS and cause cell death *via* apoptosis and necrosis (Marrache et al., 2013).

Immunotherapy can boost the protective immune responses and emerge as a promising treatment in cancer (Topalian et al., 2015). On the one hand, immunotherapy can harness the immune system to achieve an anticancer effect. On the other hand, it engendered a long-term memory effect and had characteristics of anti-relapse. However, immunotherapy of cancer faces challenges of having low tumor immunogenicity and an immunosuppressive tumor microenvironment (Turley et al., 2015). Dendritic cell (DC)-based cancer immunotherapy was also limited by the low potency of generating tumor antigen-specific T cell responses. Marrache et al. (2013) demonstrated that mitochondria-targeted nanoparticle-based light-activated breast cancer cell antigens have the potency of stimulating DCs for cancer immunotherapy (Figure 7).

Mitochondrial antigen presentation was considered as a reason for autoimmune disease development. Matheoud et al. (2016) showed that Parkin and Pink 1 proteins are in adaptive immune responses and demonstrated autoimmune mechanisms to be possible which involved Parkinson disease (PD) antigen presentation. This finding was the first to link a neurodegenerative disease like PD to autoimmunity. Voo et al. gave a mitochondrial immune target of CD4^+^ T cells which expanded from a melanoma patient. By high-dose IL-2 from this patient, the tumor-infiltrating T cells can be expanded, demonstrating a peptide which translated from another open reading frame of the mitochondrial cytochrome *b* (cyt*b*) (Yang et al., 2014). Pierini et al. established a cancer vaccine which was based on using aberrant mitochondrial protein and isolating it from the tumor as an important immunotherapeutic strategy (Yang et al., 2014; Pierini et al., 2015). It was the first vaccine which based on mtDNA-mutated peptides and derived from tumor cells that induced an immune response.

All these studies indicated cancer patients who bear mutations in mitochondrial DNA. Tumor-associated mitochondrial antigens meet the criteria of an ideal tumor-associated antigen (Pustylnikov et al., 2018). The implementation of the immune system as the mechanism in targeting unhealthy mitochondria within cancer cells attracts researchers’ interest.

### Mitochondria-Targeted Nanocarriers in sonodynamic Therapy

Sonodynamic therapy (SDT) is an excellent treatment for cancer; it utilizes ultrasound (US) irradiation and sonosensitizers to damage cancer cells (Qian et al., 2016; Pan et al., 2018; Zhu et al., 2018b). SDT is able to target the zones of lesion precisely and thus will not damage surrounding normal tissues at the same time (Qian et al., 2016; Pan et al., 2018; Zhu et al., 2018b). Ultrasound is a cheap method with a non-radioactive stimulus mechanical wave and has mini-invasiveness and deep penetration of tissue. Sonosensitizers can transfer energy upon a high-energy input to oxygen molecules and then generate reactive oxygen species (ROS) subsequently, leading to further cytotoxicity for therapeutic purposes (Chen et al., 2014). What is more, US can directly induce cancer cell apoptosis itself (Qian et al., 2016).

In cancer therapy, one of the most difficult concerns for nanomedicine is the accumulation of nanovesicles and selective localization in the tumor area (Kim et al., 2018; Mura et al., 2013). The critical part of the process is the diffusion of nanovesicles from the surface of cancer areas which could be reached from blood vessels to poorly perfused inside core areas (Mura et al., 2013; Wong et al., 2011; Liu et al., 2015; Kim et al., 2018). Nanoparticles with size up to 400 nm accumulate in tumors passively through an EPR effect, resulting from the specific leaky structure of tumor vasculature (Bae and Park, 2011; Chauhan et al., 2012).

Ultrasound combined with drug-loaded microbubbles (MBs) has been studied for improving drug delivery efficiency (Chertok et al., 2016; Ho and Yeh, 2017). It was found that MBs had a short lifespan *in vivo*, thus restricting the duration of therapeutic effects (Ho and Yeh, 2017). Upon ultrasound irradiation, acoustic nanodroplets (NDs) with liquid cores can transform into MBs. This process is called acoustic droplet vaporization (ADV), creating a non-demand production of MBs, vascular disruption, and tissue erosion (Kagan et al., 2012; Mura et al., 2013; Ho and Yeh, 2017). Some ligands for active targeting can be integrated into nanovesicles and can help improve the therapeutic efficacy of cancer cells (Zhao et al., 2018; Zhu et al., 2018a). Mitochondria-targeting drugs can explore the susceptibility of mitochondria to ROS (Li et al., 2013; Yang et al., 2016; Zielonka et al., 2017; Wang et al., 2018). PDT demonstrated successfully in some preliminary works, as shown in previous studies (Jung et al., 2017; Noh et al., 2018). Thus, SDT is also believed to be effective when including mitochondria-targeted sensitizers (Shimamura et al., 2016).

**FIGURE 8:**
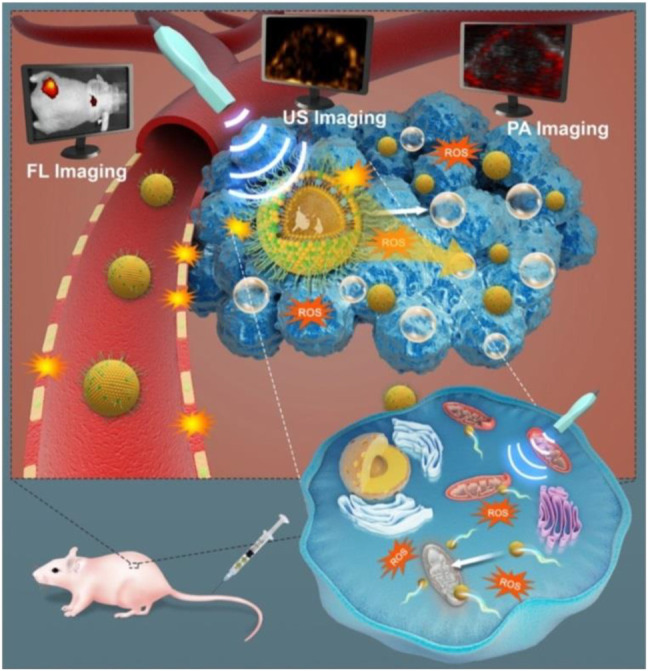
As-synthesized US-responsive NDs for efficient SDT which include deep penetration, tumor cell- or mitochondria-targeting ability, ADV, and guidance or monitoring by multimodal (US, PA, and FL) imaging (Zhang et al., 2019).

Zhang et al. (2019) found that IR780-NDs which were US-activated NDs with a core/shell structure were constructed with enhancing deep penetration mitochondrial targeting and for SDT with concurrent FL/US/PA imaging guidance. The NDs accumulate in the area of cancer from the circulation system of blood through the EPR effect. Because of the susceptibility of mitochondria toward ROS, the inherent mitochondria-targeting capability can further increase the ROS cytotoxicity during the SDT process. Through US irradiation, ADV occurs, that is, acoustic NDs transfer into MBs. ADV induces tissue erosion and vascular disruption, thus allowing much more droplets to leave the systemic circulation and enter the tumor stroma, then penetrate into the inner tissues, which are farther from the blood vessels. Loading with IR780, the diffusion of NDs to deeper tumor could be assisted. Therefore, IR780-NDs combined with is a promising theranostic nanoplatform for cancer therapy (Figure 8) (Zhang et al., 2019).

## Conclusion and Perspectives

Mitochondria are essential organelles for ATP generation and are the center of cell death regulation. The functions between normal cells and cancer cells of mitochondria are different. Thus, it may offer the potential for designing anticancer agents which can deliver mitochondrial targeting drugs to kill cancer cells selectively. Targeting the mitochondrial delivery of anticancer drugs plays an important role in diseases in recent decades. Cancer stem cells have unique characteristics which make them much vulnerable to mitochondria-targeting drugs like some natural compounds. Thus, identifying mitochondria-targeting drugs from various natural substances presents a promising way for further research.

Nanostructures have the potential for delivering drugs; however, due to various biological barriers of nanomedicines, clinical applications are in the early stage and the efficacy is limited. The combination of nanostructure or stimulus responsiveness with a desired mitochondria-targeted drug-free strategy can greatly enhance the efficacy in treating cancer. Here in this review, we described how these mitochondria-targeted nanocarriers promote highly efficient cancer treatment in PDT, chemotherapy, combined immunotherapy, and SDT. Cancers are very complex; a single drug or single therapy sometimes may not be enough to treat tumor. Combined treatment such as PDT, immunotherapy, and others should be applied to fight against with cancer. Thus, multiple mitochondria-targeted nanosystems are needed for combined therapy.

Although mitochondria-targeted nanocarriers have achieved great progress, there are many key questions that still remain unsolved, for example biosafety, the solubility of nanocarriers, targeting, penetration of tumors, uptake and retention in reticuloendothelial organs, and long-term fate. We believe that mitochondria-targeted nanocarriers can help with treatment of not only cancer but also other diseases such as neurological diseases. The clinical applications of various mitochondria-targeted nanocarriers still need more efforts. The designs and construction of mitochondria-targeted nanocarriers are also a critical challenge.
